# Antibacterial consumption in public health facilities in Uganda: a trend analysis of national warehouse distribution data 2022–2024

**DOI:** 10.1093/jacamr/dlag045

**Published:** 2026-04-09

**Authors:** Benard Nsubuga, Ventrine Marion Chelimo, Aaron Kayemba, Philip Ampaire, Winnie Nambatya, Kalidi Rajab, Vivian Twemanye, Harriet Akello, Anthony Ddamba, Moses Kamabare, David Arinaitwe, Francis Kakooza, Richard Walwema

**Affiliations:** Department of Client Services, National Medical Stores, Entebbe, Uganda; Department of Client Services, National Medical Stores, Entebbe, Uganda; Department of Client Services, National Medical Stores, Entebbe, Uganda; Department of Client Services, National Medical Stores, Entebbe, Uganda; Department of Pharmacy, School of Health Sciences, College of Health Sciences, Makerere University, University Rd., Kampala 10218, Uganda; Department of Pharmacy, School of Health Sciences, College of Health Sciences, Makerere University, University Rd., Kampala 10218, Uganda; Global Health Security, Infectious Diseases Institute, Kampala, Uganda; Department of Pharmaceuticals and Natural Medicines, Ministry of Health, Kampala, Uganda; Department of Client Services, National Medical Stores, Entebbe, Uganda; Department of Client Services, National Medical Stores, Entebbe, Uganda; Department of Client Services, National Medical Stores, Entebbe, Uganda; Global Health Security, Infectious Diseases Institute, Kampala, Uganda; Global Health Security, Infectious Diseases Institute, Kampala, Uganda

## Abstract

**Background:**

While multiple factors contribute to antimicrobial resistance (AMR), the consumption of antimicrobials is a major modifiable cause. Therefore, accurate understanding of antibacterial exposure is critical for effective stewardship.

**Objective:**

This study analysed trends of antibacterial distribution data in Uganda between 2022 and 2024.

**Methods:**

This was a retrospective cross-sectional study that used data on antimicrobials that were distributed to government-owned health facilities between 2022 and 2024. Data analysis used the WHO Global Antimicrobial Resistance and Use Surveillance System antimicrobial use methodology that uses anatomical therapeutic chemical classification and defined daily doses (DDD) system to classify antimicrobials to estimate antibiotic use volumes.

**Results:**

The study revealed an upward trend in antibacterial consumption (ABC) from 2.88 DDD per 1000 inhabitants per day (DID) in 2022 to 10.21 DIDs in 2024. The highest consumption was observed in primary healthcare facilities. The most consumed antibiotics across the three years were penicillin (amoxicillin) (28% in 2022, 24% in 2023 and 37% in 2024) and sulfamethoxazole and trimethoprim (cotrimoxazole) (26% in 2022, 34% in 2023 and 13% in 2024). For the AWaRe classification, antibiotics in Access group (84.1% in 2022, 88.6% in 2023 and 87.0% in 2024) had the largest percentage of DIDs.

**Conclusions:**

The study established a marked increase in ABC within Uganda’s public sector between 2022 and 2023, followed by a modest rise in 2024. The rise in resistance to commonly used agents could sustain higher demand for some antibiotics. These findings underscore the importance of linking distribution data with facility-level prescribing and resistance patterns to inform stewardship interventions.

## Background

Bacterial infections are increasingly becoming a major public health concern all over the world.^1–3^ Globally, it is estimated that ∼1.27 million deaths were attributed to antibiotic resistant bacterial infections in 2019 with one in every five deaths occurring in children below 5 years, while in 2023, one in every six bacterial infections were due to bacteria resistance to antibiotics.^3,4^ Recent estimates by the Global Research on Antimicrobial Resistance Project, using 2022 data, indicated that bacterial AMR was associated with 4.95 million deaths globally, with projections suggesting a substantial increase by 2050 if current trends persist.^5^ The misuse and overuse of antibiotics across human, animal and environmental sectors are key drivers of resistance among bacterial pathogens.^6–8^ The burden of antibiotics misuse is multifaceted, rooted in inappropriate indication, improper selection of antibiotics, inappropriate quality, dosage and dispensing among others.^6^ The burden of bacterial resistant pathogens consequently deepens both health and financial strain due to hospital long stays and reduced labour productivity among humans.^1^ Globally, monitoring of antibiotics consumption is at the forefront of many healthcare systems.^3^ Over the years, WHO has supported the development and strengthening of national surveillance systems to harmonize and report on antimicrobial use (AMU) to foster appropriate use of antimicrobials including antibacterials.^3,9^ The WHO provides a platform to monitor the consumption of antibacterials through the Global Antimicrobial Surveillances Systems or Antimicrobial Use (GLASS-AMU).^9^ This provides the international and country level prevailing trends in antimicrobial consumption (AMC) to understand consumption patterns aimed at facilitating development of stewardship programmes.

Uganda launched the country’s second national action plan on AMR prioritizing surveillance and optimization of AMU across human, and agricultural sectors through a One Health approach.^10^ These efforts aim to preserve the effectiveness and efficacy of antimicrobial agents for human and animal health through controlled access, effective AMS and appropriate use. In addition, the WHO’s AWaRe (Access, Watch and Reserve) categorization, introduced in 2017, supports stewardship by categorizing antibiotics into Access, Watch and Reserve groups based on their spectrum of activity, potential for resistance and importance in human medicine to guide monitoring and promote appropriate use.^11^ Therefore, surveillance of AMC and AMU is an important action in guiding AMS activities, to measure existing practices, identify and prioritize and investigate problems, and monitor effects of interventions.^12^ However, published trends of AMC are limited to high-income countries and gaps remain in understanding the AMC trends in developing economies including Uganda.^13,14^ Available data on consumption of antimicrobials in Uganda are fragmented across different programmes limiting the ability to generate complete national picture and guide rational AMU. The available national-level studies on AMC in Uganda either used imported data on antimicrobials with a lack of emphasis on antibacterials, lacked differentiating public and private health sector consumption and covering a subset of healthcare facilities that thereby lacked generalizability, particularly for the public sector in Uganda.^15,16^ Although previous work has examined antibiotic distribution from central warehouses to inpatient care facilities in Uganda for 2017–2019, comprehensive analyses covering more recent post-pandemic years are lacking. This study addresses that gap by providing a national-level assessment of ABC across all government-owned health facilities using national warehouse distribution data for 2022–2024 to support evidence-based interventions and implementation of effective AMS strategies that contribute to the control of AMR.^16^

## Methods

### Study design

This was a retrospective cross-sectional study that used National Medical Stores (NMS) data from the distribution of essential medicines and health supplies (EMHS) across various levels of care, spanning three calendar years (2022, 2023 and 2024). AMC is defined as the quantity of antimicrobials used by a population in a specific setting (e.g. community or hospital health care level) during a specific period (e.g. days, months and years).^17^ In the context of this study, ABC data refers to estimates derived from aggregated data from NMS, which contains no details on the patients who received the medicines or indications for antibacterial prescriptions. These data sources provide a proxy estimate for the use of antibacterial.^9,17^

### Study setting

Uganda is a landlocked country located in Eastern Africa. Uganda's population as of May 2024 was 45 905 417 people with an average annual growth rate of 2.9%.^18^ The study was carried out at the NMS, a government-owned organization mandated to procure, store and distribute EMHS to all government-owned HFs.

Health services in Uganda are provided by both public (government-owned) and private actors. Health care in the public sector is delivered through a referral system that consists of national referral hospitals (NRH) and national referral institutes (NRIs), regional referral hospitals (RRHs), general hospitals (GHs), health centre IVs (HC4s), health centre IIIs (HC3s) and health centre IIs (HC2s). NRHs and NRIs provide highly specialized medical services and serve as the apex referral institutions for all RRHs. RRHs deliver specialized care at the regional level and act as referral centres for lower-tier facilities, primarily GHs within their respective catchment areas. GHs, often referred to as district hospitals, offer general medical services within their administrative districts. HC4s were created to provide essential healthcare to populations within county-level administrative units including provision of emergency surgery and blood transfusion services and all other health care services offered at HCIIIs.^19^ HCIIIs are situated at subcounty level and provide maternity services, outpatient health care services, inpatient health care services and laboratory services, while HCIIs are situated at parish level to offer outpatient health care, antenatal health care, immunization services and conduct outreach activities.^19^ NMS distributes medicines to all government-owned HFs comprising five NRHs, two NRIs, 18 RRHs, 52 GHs, 208 HCIVs, 1279 HCIIIs and 1762 HCIIs. NMS is mandated to procure, store and distribute EMHS to all public health facilities in the country. NMS accounts for 100% of the antibacterials supplied to government-owned HFs.

### Data sources

The study involved analysis of routinely generated EMHS distribution data. The data were extracted from the NMS+^®^ database, an enterprise resource planning system used by the NMS to manage receipt of stock into the warehouse, warehousing processes, receipt of HF orders and issuing stock to individual HFs. Detailed information about antibacterial medicines, including their strength, dosage form, pack size and total quantity issued are all captured in the database.

### Eligibility criteria

This study focused on antibacterials comprising antibacterials for systemic use [anatomical therapeutic chemical (ATC) code J01] and drugs for treatment of tuberculosis (J04A).^17^

### Data collection

**Figure 1.**
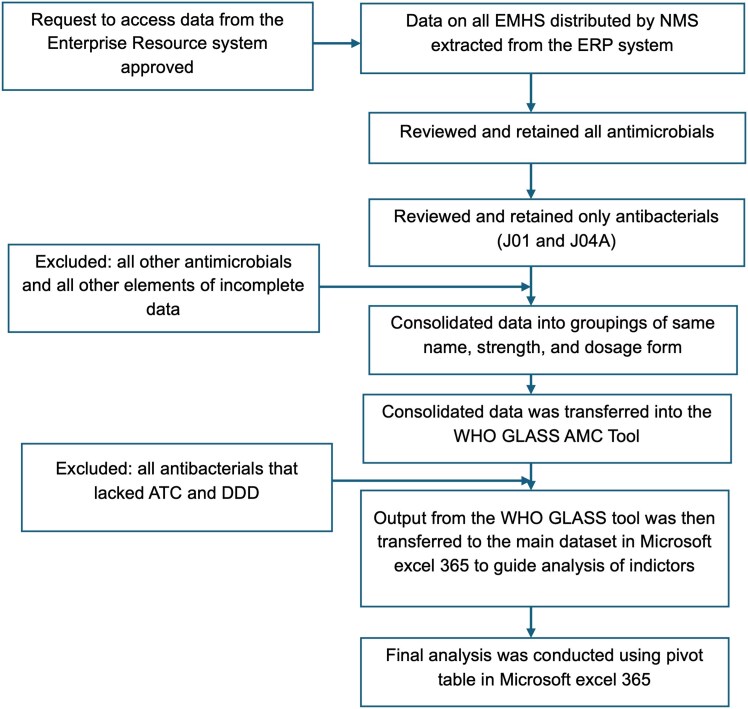
ABC data mining, cleaning and preliminary analysis.

The WHO GLASS methodology for AMC was adapted to suit the NMS distribution dataset. The data were extracted from the NMS+ database for the years 2022 to 2024 and were exported to Microsoft Excel for analysis. The data were then filtered to include antibacterials for systemic use and antituberculosis agents. Non-J01 antimicrobial products, incomplete records and duplicates were excluded. The variables extracted included year of distribution, name and level of care of the recipient facility, unique product code, product name, generic name, product strength, unit of measure, route of administration, pack size and number of packs. Each product was assigned an ATC code and corresponding defined daily dose (DDD) value using the WHO ATC/DDD Index 2022. Different DDDs were considered for different formulations of the same drug since the bioavailability may be substantially different for various routes of administration.^17^ Pack sizes were converted to total quantities distributed using the product’s strength, unit, and number of packs issued. The cleaned data were aggregated by year and by level of care (HCII–NRH), and population denominators were obtained from the Uganda Bureau of Statistics (UBOS) 2021 census projection. Figure 1 summarizes the process of data mining and cleaning.

### Data analysis

Estimates of ABC were expressed as DDD per 1000 inhabitants per day abbreviated to DID using the standard WHO formula.^17^ The 2021 population of Uganda (42 885 900) was used as the denominator to calculate consumption for all the years. The study calculated DIDs by ATC classes for all antibiotic class, AWaRe classification, period of study, level of care and route of administration (RO).

The total DDDs per package were calculated using the formula:


(1) $$DDD=\frac{SxPxTQI}{Assigned\;of\;the\;antimicrobial\;DDD}$$

where DDD is total DDD per package; *S* is the strength (g or mg); *P* is the unit of measure and TQI is the total quantity issued.

The DID was then calculated as:


(2) $$DID=\left(\frac{DDD\times 1000}{(365\times Total\;Population}\right)$$

The consumption data were summarized using descriptive statistics including the mean, totals and percentages presented in tables and graphs. Trends in ABC were established for 3 years using DDDs per package, total volume in DDDs, and DID. This study used Microsoft Excel 365 to conduct data analysis.

In this study we used two methods to derive and illustrate the trend in our data points for the three years under study. First, we used a graphical illustration of the data points. Second, we applied a simple linear regression (using least square estimation method) to fit a linear trend line, treating ABC as the dependent variable and time in years as the independent variable. This study used 3 years (data points) to establish the trend line. Whereas 3 years may not be statistically sufficient, this is not far from the same time scope that was used by the European Centre for Disease Prevention and Control that used 5 years (2019–2023) in a linear regression model to establish trends in AMC in EU.^14^

### Ethical considerations

The Helinski ethical principles require all studies involving human participants to protect their rights, including those of patients and healthy volunteers.^20^ This study, however, did not involve human participants, animals or identifiable human material. This study used aggregated anonymized secondary data on antibacterials distribution from a central warehouse. Permission to access secondary data from the central warehouse was sought from NMS’ management. The general manager of NMS granted permission to access and use the AMC data. Finally, the study was exempt from ethical review by the Makerere University School of Health Sciences Research and Ethics Committee.

## Results

### Trend of total ABC (DID) per year

**Figure 2.**
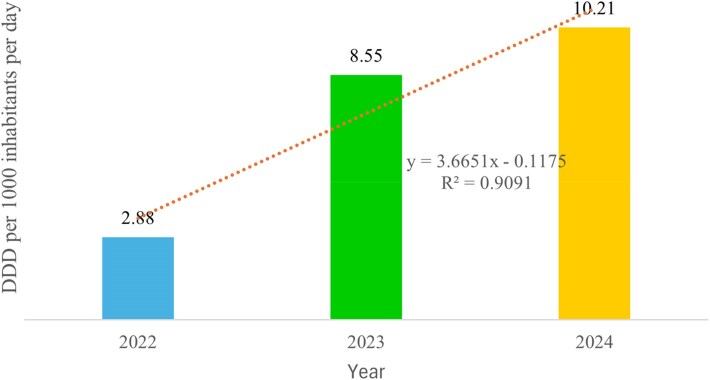
Trend of total ABC for the years 2022–2024, expressed as DDD per 1000 inhibitors per day (DID).

In this study, there was an increase in ABC in the public health sector in Uganda from 2.88 DIDs in 2022 to 10.21 DIDs in 2024. The findings portray an upward trend in the 3 years under review. Consumption of antibacterials in 2023 (DID = 8.55) and 2024 (DID = 10.21) was on average four times higher compared with 2022 (DID = 2.88). The ABC in 2022 increased by 197% (DID = 5.67/2.88) compared with 2023 while that of 2023 increased by only 19% (DID = 1.66/8.55) compared with 2024. The ABC data depicted a linear upward trend where, any additional year from 2022 onwards increased antibacterial use in the public sector by 3.66 DIDs. In addition, a linear trend was substantially suitable for the ABC data $\left(R^{2}=0.91\right)$. This implied that 91% variation in the ABC could be explained by the time factor in the model and 9% variation was due to unobserved characteristics. (Figure 2)

### Antibacterial consumption (DID) at each level of care per year

**Figure 3.**
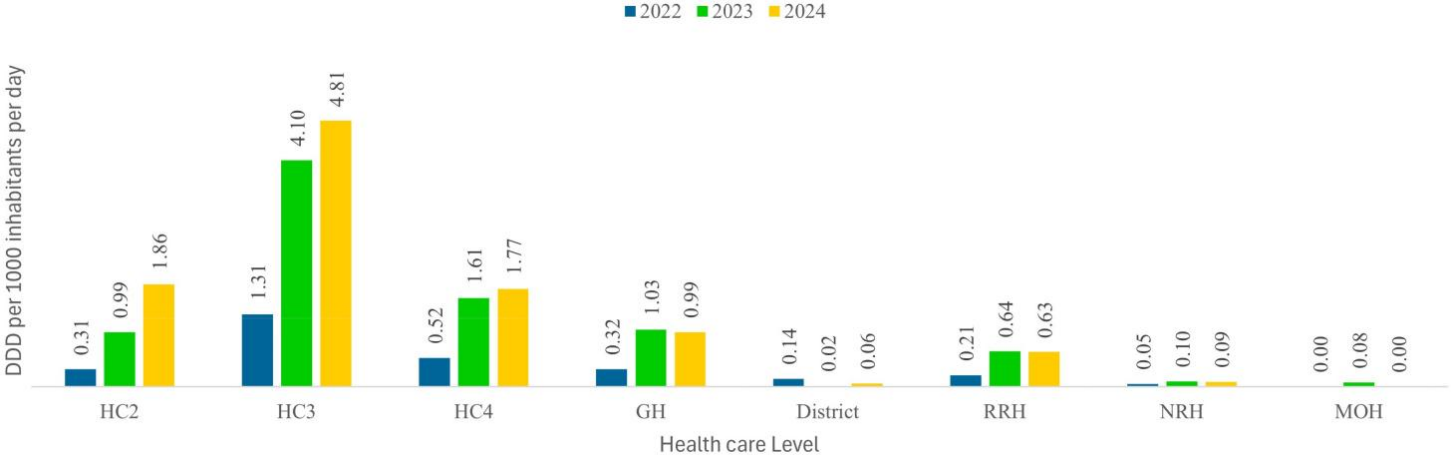
Trends in ABC expressed as DDD per 1000 inhibitors per day (DID) and stratified by level of care.

There was a general increase in ABC across the lower levels of care (HC2, HC3, HC4) between 2022 (DID = 2.15) and 2024 (DID = 8.44). The cumulative ABC in HC3 level was highest across the 3 years. However, there was a slight decline in ABC for the higher levels of care (GH, RRH, NRH) from 2023 (DID = 1.77) to 2024 (DID = 1.71). The DID was highest in HCIIIs and HCIVs because of the high composition of distribution sites at these levels of care. (Figure 3)

### Antibacterial consumption by AWaRe classification

**Figure 4.**
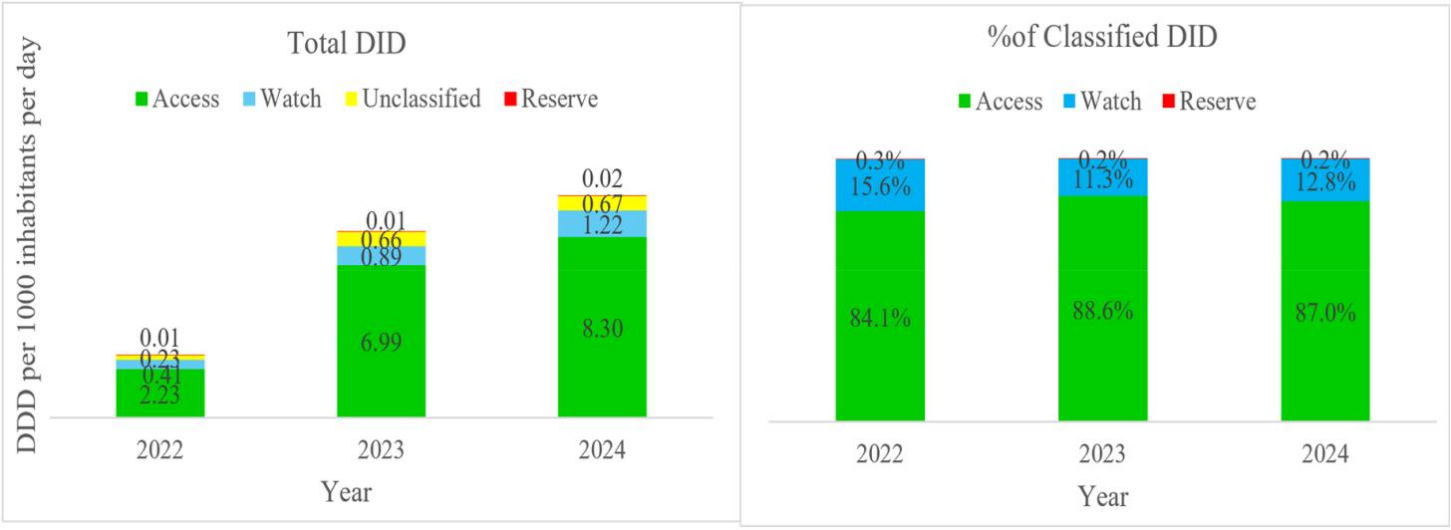
ABC expressed as DDD per 1000 inhibitors per day (DID) and by AWaRe Classification.

The findings indicated that, overall, antibacterials in the Access class had the highest consumption from 2022 to 2024, accounting for 84.1% (DID = 2.23) in 2022, 88.6% (DID = 6.99) in 2023% and 87.0% (DID = 8.30) in 2024 in the classified antibacterials. The composition of unclassified antibacterials includes mostly Anti TB and are not classified as per the WHO 2023 AWaRe classification of antibiotics. (Figure 4)

### Antibiotics consumption by AWaRe classification antibacterials that contributed <75% of DIDs consumed per year

Amoxicillin contributed the highest DIDs in 2022 (28.0%) and 2024 (37.0%), while sulfamethoxazole and trimethoprim contributed the highest DIDs in 2023 (34.0%). A total of five items namely: amoxicillin, sulfamethoxazole and trimethoprim, metronidazole, doxycycline and ciprofloxacin were highly consumed throughout the 3 years. Four items were consistently the top contributors to the DIDs in the 3 years (amoxicillin, sulfamethoxazole and trimethoprim, metronidazole and doxycycline). (Table 1)

**Table 1.** Antibacterials that contribute under 75% of DIDs consumed per year

DIDs per 1000 people per day							
Chemical substance	2022		2023		2024		AWaRe
	AMC	(%)	AMC	(%)	AMC	(%)	
amoxicillin	0.80	28	2.08	24	3.83	37	Access
sulfamethoxazole and trimethoprim	0.74	26	2.92	34	1.36	13	Access
metronidazole	0.30	10	0.82	10	1.22	12	Access
doxycycline	0.27	9	0.72	8	1.23	12	Access
ciprofloxacin	0.17	6					Watch
Total AMC under 75%	2.16		6.41		7.66		

### ABC via route of administration

**Figure 5.**
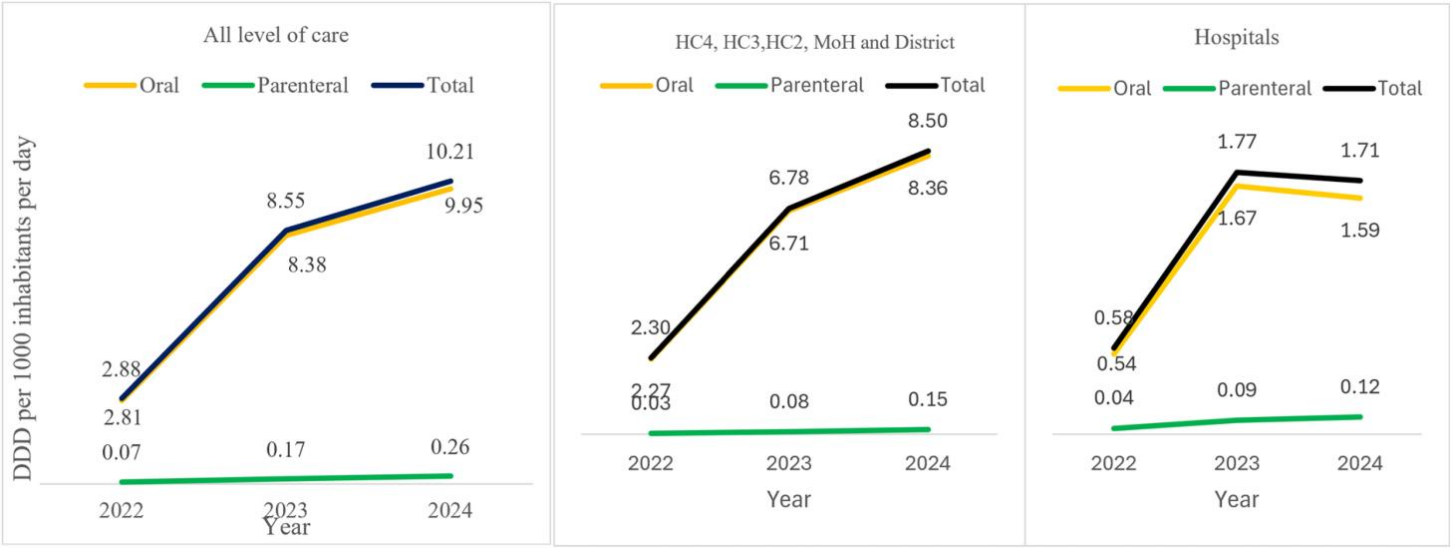
ABC by route of administration expressed as DDD per 1000 inhibitors per day (DID).

The ABC by oral administration accounted for 97.6% (DID = 2.81/2.88), 98.0% (DID = 8.38/8.55) and 97.4% (DID = 9.95/10.21) of the total ABC in 2022, 2023 and 2024 respectively. On the other hand, a similar consumption pattern was observed in the ABC for parenteral antibacterials in 2022 (2.4%, DID = 0.07/2.88), 2023 (2.0%, DID = 0.17/8.55) and 2024 (2.6%, DID = 0.26/10.21). (Figure 5)

### Consumption of antibacterials for systemic use (J01, ATC3)

Beta-lactam (penicillins) were the highest consumed antibacterials in 2022 (36.2%, DID = 0.845) and 2024 (48.1%, DID = 3.985) while sulphonamides and trimethoprim class antibacterials (41.5%, DID = 2.921) were the highest consumed in 2023. Overall, beta-lactam antibacterials, sulphonamides and trimethoprim, tetracyclines and quinolones were the highest consumed classes across 3 years. (Table 2)

**Table 2.** Consumption of antibacterials for systemic use expressed as DDD per 1000 inhibitors per day (DID)

Description	2022		2023		2024	
	AMC	(%)	AMC	(%)	AMC	(%)
Aminoglycoside antibacterials	0.0125	0.5	0.0331	0.5	0.0470	0.6
Amphenicols	0.0007	0.0	0.0027	0.0	0.0034	0.0
Beta-lactam antibacterials, penicillins	0.8446	36.2	2.1949	31.2	3.9852	48.1
Macrolides, lincosamides and streptogramins	0.1949	8.4	0.2936	4.2	0.3525	4.3
Other antibacterials	0.0580	2.5	0.2666	3.8	0.4310	5.2
Other beta-lactam antibacterials	0.0416	1.8	0.0987	1.4	0.1629	2.0
Quinolone antibacterials	0.1775	7.6	0.4959	7.1	0.7084	8.6
Sulfonamides and Trimethoprim	0.7353	31.5	2.9207	41.5	1.3584	16.4
Tetracyclines	0.2672	11.5	0.7248	10.3	1.2319	14.9
Total	2.3322	100.0	7.0310	100.0	8.2807	100.0

## Discussion

This study analysed the trends in consumption of antibacterials in Uganda in the years 2022, 2023 and 2024, with a focus on the pattern of ABC observed through the distribution of different antibacterials and AWaRe classification.

The ABC increased by >255% between 2022 and 2024 while the range of antibacterials used did not change. The DIDs (2.88) seen in 2022 were much lower than in 2023 (8.55) and 2024 (10.21). This could be attributed to disruptions that were observed in the distribution of essential health commodities to public HFs that emanated from after the COVID-19 pandemic in 2022.^21^ In addition, the increased funding by the government of Uganda in 2023 and 2024 towards the supply of medicines in public HFs may have bloated the supply of antibacterials in the public sector landscape.^22^ This is coupled with debilitated selection and planning for antibacterials, where the use of antibiograms in selection and planning for antibiotics is inadequate. An antibiogram is an essential tool developed to track any changes in antibiotics and to guide empirical antibiotics therapy.^23^  *A priori*, each HF with sound laboratory infrastructure in Uganda ought to develop an institutional antibiogram not only to influence prescription practices but also guide the selection of antibiotics suitable for use within their geographical setting.^24^

Whereas this study analysed antibiotics aggregate distribution data from the national warehouse, the selection and consumption for such antibiotics happens at subnational level within the HFs. The upward trend observed in the use of antibiotics could be attributed to inappropriate prescription practices, pressure exerted on health care providers to prescribe antibiotics including conditions that lack warrant to prescribe antibiotics, insufficient diagnostic tools and limited adherence to treatment guidelines.^25,26^ For example, Nuwematsiko *et al.* in their study to establish the prevalence of antibiotic prescriptions in two selected districts in Uganda, found that three in every four antibiotics were inappropriately prescribed while Obakiro *et al.* in a related study found 82.6% non-adherence to treatment guidelines when prescribing antibiotics, a problem that was more pronounced in the treatment of bacterial pathogens in children under 12 years of age.^25,26^ Similarly, inappropriate use of antibiotics is prevalent in other LMICs such as Kenya and Nigeria.^27,28^ The increased inappropriate use of antibacterials treacherously burdens the health care system including misalignment of resources when procuring medicines.

Generally, ABC trends analysed in this study at the national level may not be comparable to similar studies elsewhere, few studies in recent time have focused their analysis on ABC trends only using distribution aggregate data. Similar studies in Uganda, Ethiopia and South Africa all tackled antimicrobials holistically.^16,29,30^

This study revealed that in Uganda, penicillin (Amoxicillin) (DID = 0.80 in 2022, DID = 2.08 in 2023 and DID = 3.83 in 2024) was the most consumed followed by sulfamethoxazole and trimethoprim (Cotrimoxazole) (DID = 0.74 in 2022, DID = 2.92 in 2023 and DID = 1.36 in 2024). This is attributed to their use in national programmes. For example, amoxicillin is widely used in the integrated community case management programme under the village health team structure while Cotrimoxazole on the other hand, is used in the prevention of opportunistic infections among patients with HIV, which is of a high burden in Uganda. This finding is consistent with Murungi *et al.* (2023) who found increased use of penicillins and sulfamethoxazole and trimethoprim in Uganda between 2018 and 2022.^15^ Whereas the current study focused on public sector ABC as established through distributional data of public HFs, Murungi *et al.* (2023) used import data as a proxy and covered all antimicrobials.

The study revealed that the most consumed antibiotics in the public health sector in Uganda belonged to the Access category (84.1% in 2022, 88.6% in 2023 and 87.0% in 2024), a finding similar to most other countries globally according to the WHO 2022 GLASS report.^31^ The discovery of antibiotics has been a momentous achievement in the twentieth century which accelerated changes in medical practice and drastically combated morbidity and mortality associated with bacterial infections.^32^ However, the inappropriate consumption of antibiotics, sometimes mirrored through high consumption patterns, is strongly associated with increased frequency of inappropriate antibiotic use.^32,33^ As such, WHO developed an internationally acceptable classification, the AWaRe categorization, to guide access and use of antibiotics to treat common infections.^34^ Antibiotics in the Access category are those that have activity against a wide range of commonly encountered susceptible pathogens while also showing lower resistance potential than antibiotics in the other groups.^34^ WHO recommends that national-level consumption of antibacterials in the Access category should be 60% or more.^35,36^ However, this was later revised to 70% in November 2022 by the Third Global High-level Ministerial Conference on AMR in Muscat, Oman.^2,31^ The trend in the distribution of antibiotics in the public sector in Uganda depicts better use of antibiotics in the Access group compared with other LMICs such as South Africa.^29,30^ In South Africa, between 2018 and 2022, antibiotics in the Watch group (52%) were the most used followed by Access (48%).^30^ However, Malaysia reported a 90% antibiotic use in the Access group between 2018 and 2021 for the public sector, slightly higher than our findings for Uganda.^37^ The study in Malaysia was conducted at primary healthcare points where access to Watch and Reserve category antibiotics may be limited. Generally, high consumption of Access antibiotics is considered desirable since these are the most recommended medicines as first line or second line treatment for common infections and are less prone to driving AMR compared with Watch and Reserve antibiotics. Therefore, the national AMR control programmes should ensure that AWaRE classification is systematically integrated into national treatment guidelines and the AMS strategies to sustain the observed trend in the use of antibiotics in the Access class. It is also important to note that even though the percentage consumption of Watch and Reserve antibacterials compared with the Access class has not changed significantly, their actual consumption in DIDs has been increasing annually. Therefore, it is essential to have AMS programmes with targeted interventions to ensure appropriate use of the Watch and Reserve classes of antibiotics.

This study was not conducted without limitations. The study results may not be generalizable to the entire health sector. The study focused on the public sector without necessarily tackling comparison with the private sector. However, public sector serves most of the population in Uganda and may therefore reflect a country situation. In addition, distribution data from the procurement and distribution agent for Uganda was used as a proxy for national-level public sector antibacterial use. This may not truly reflect the actual antibacterial use at service delivery points. Finally, the term AMC used to mean estimates from aggregate national-level data is now synonymous with AMU that is used to refer to antimicrobial data from end use points at subnational level.^31^

### Conclusion

The study found a marked increase in ABC within Uganda’s public sector between 2022 and 2023, followed by a more modest rise in 2024. This sharp initial increase was probably influenced by post-COVID-19 supply recovery and increased government funding for essential medicines, while the slower growth thereafter may indicate a stabilization of supply or improved rationalization in distribution. In the next 2 or 3 years, ABC may plateau if current stewardship and procurement optimization efforts take hold. However, a rise in resistance to commonly used agents could sustain higher demand for some antibiotics. These findings underscore the importance of linking supply data with facility-level prescribing and resistance patterns to inform targeted stewardship interventions.

## Data Availability

Raw data will be available upon reasonable request.
